# Viral sensing by epithelial cells involves PKR- and caspase-3-dependent generation of gasdermin E pores

**DOI:** 10.1016/j.isci.2023.107698

**Published:** 2023-08-21

**Authors:** Coralie Guy, Marcin Baran, Pau Ribó-Molina, Bernadette G. van den Hoogen, Andrew G. Bowie

**Affiliations:** 1 School of Biochemistry and Immunology, Trinity Biomedical Sciences Institute, Trinity College Dublin, Dublin 2, Ireland; 2 Department of Viroscience, Erasmus Medical Center, Rotterdam, the Netherlands

**Keywords:** Immunology, Virology

## Abstract

Viral sensing in myeloid cells involves inflammasome activation leading to gasdermin pore formation, cytokine release, and cell death. However, less is known about viral sensing in barrier epithelial cells, which are critical to the innate immune response to RNA viruses. Here, we show that poly(I:C), a mimic of viral dsRNA, is sensed by NLRP1 in human bronchial epithelial cells, leading to inflammasome-dependent gasdermin D (GSDMD) pore formation via caspase-1. DsRNA also stimulated a parallel sensing pathway via PKR which activated caspase-3 to cleave gasdermin E (GSDME) to form active pores. Influenza A virus (IAV) infection of cells caused GSDME activation, cytokine release, and cell death, in a PKR-dependent but NLRP1-independent manner, involving caspase-8 and caspase-3. Suppression of GSDMD and GSDME expression increased IAV replication. These data clarify mechanisms of gasdermin cleavage in response to viral sensing and reveal that gasdermin pore formation is intrinsically antiviral in human epithelial cells.

## Highlights

• Epithelial cell cytokine release and cell death by dsRNA are gasdermin dependent
• Gasdermin D pores are NLRP1 dependent while gasdermin E requires PKR and caspase-3
• Influenza A virus activates the PKR-caspase-3-gasdermin E pathway but not NLRP1
• Epithelial cell gasdermin D and E pores are antiviral for influenza A virus

## Graphical abstract

**Figure:**
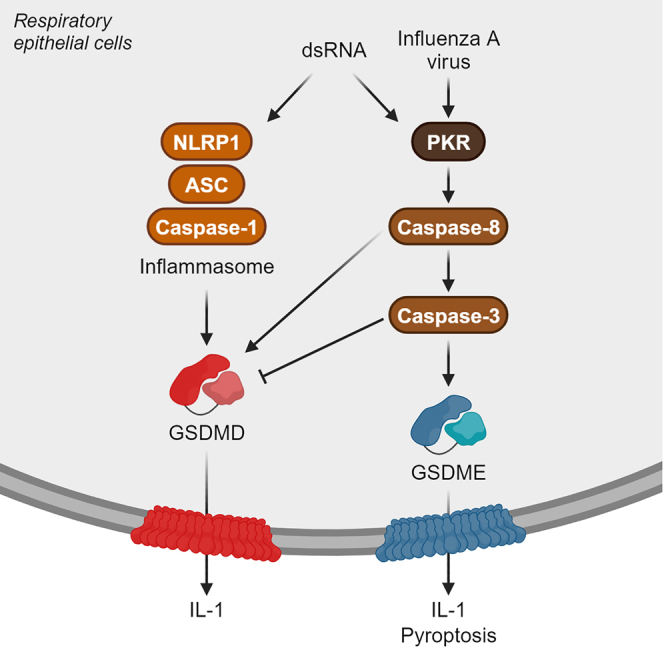

## Introduction

The initiation of innate immune responses to pathogens involves cell surface and intracellular sensor proteins detecting the presence of pathogen-associated molecular patterns (PAMPs) and danger-associated molecular patterns (DAMPs) that indicate altered cellular homeostasis. These sensors can be largely broken down into two families – pattern recognition receptors (PRRs) which when activated signal altered gene expression, for example the induction of cytokines and interferons, and inflammasomes, which activate caspase-1 to drive pro-inflammatory cytokine secretion and pyroptosis.

In the case of viral pathogens, viral and mislocalized host nucleic acids are key PAMPs and DAMPs, respectively, and antiviral PRRs include endosomal Toll-like receptors (TLRs) and cytosolic retinoic acid-inducible gene-I (RIG-I)-like receptors (RLRs) that detect viral RNA, and cGAS which responds to cytosolic viral and host double-stranded DNA. Antiviral inflammasomes are less well characterized than PRRs, but nucleotide-binding oligomerization domain, leucine-rich repeat and pyrin domain-containing 3 (NLRP3) has been implicated in responses to RNA viruses in myeloid cells. Upon activation, NLRP3 binds to the adaptor protein apoptosis-associated speck-like protein containing a CARD (ASC) which recruits pro-caspase-1 to form a cytosolic multiprotein complex. This leads to auto-activation of caspase-1 which then processes pro-interleukin (IL)-1β into its mature form, IL-1β, and also cleaves gasdermin D (GSDMD) to release an active p30 fragment that forms membrane pores leading to membrane permeabilization and pyroptosis.^1^^,^^2^

Thus, gasdermin family members are pore-forming proteins that mediate pro-inflammatory lytic cell death. Gasdermins possess an N-terminal (NT) pore-forming domain and a C-terminal (CT) repressor domain that are bound together by a linker region.^3^ At steady state, the CT domain interacts with the NT domain to prevent constitutive activation of gasdermins. Cleavage of GSDMD, the most characterized protein of the gasdermin family, by caspase-1, occurs in the interdomain linker, releasing the NT domain and enabling it to bind to acidic membrane phospholipids where it oligomerizes and forms pores in the plasma membrane.^2^^,^^4^ GSDMD pores range from 10 to 20 nm^5^^,^^6^ and allow the secretion of small cytokines such as IL-1β. Accumulation of GSDMD pores causes plasma membrane rupture and hence pyroptotic cell death. More recently, another member of the gasdermin family, gasdermin E (GSDME), has been shown to have a role in pyroptosis.^7^^,^^8^ GSDME is cleaved by caspase-3 in its interdomain region to release the NT domain which results in pore formation, cytokine secretion, and pyroptotic cell death.^7^^,^^8^^,^^9^ Although gasdermins have been intensively studied, not much is known about their role in innate immune responses to viral infection, especially outside the context of myeloid cells. Specifically, how inflammasomes and gasdermins might contribute to defense against RNA viruses in epithelial cells is unclear, even though such cells represent the first line of defense against respiratory RNA viruses such as severe acute respiratory syndrome coronavirus 2 (SARS-CoV-2) and influenza A virus (IAV).

IAVs remain a major threat to human health and can cause severe symptoms in young, elderly, and immunocompromised populations, leading to annual IAV epidemics which endanger between 3 and 5 million people of severe illness and cause 290,000 to 650,000 deaths worldwide.^10^ IAV is an enveloped virus containing eight single-stranded negative-sense RNA segments.^10^ IAV RNA is recognized by host PRRs such as TLRs and RLRs, and IAV infection also activates the NLRP3 inflammasome in myeloid cells.^11^ Further, RNA viruses form replicative intermediates during viral replication that can be recognized by double-stranded RNA (dsRNA)-dependent protein kinase (PKR) which mediates host translation shutoff.^12^ In addition, IAV primarily targets respiratory epithelial cells to replicate^10^ where it causes both apoptotic and pyroptotic cell death, and IL-1β release^13^ through poorly defined mechanisms.

Even though respiratory epithelial cells have originally been described as a passive barrier in the defense against pathogens, it is now incontestable that they are essential in mounting an innate immune response against viruses, including IAV.^14^ However, information on the mechanism of action of the response of epithelial cells to virus infections, and viral nucleic acid, lags behind that known for myeloid and other immune cells. In this study, we investigated the mechanism of gasdermin cleavage upon sensing of dsRNA or IAV in primary normal human bronchial epithelial (NHBE) cells. We used intracellular delivery of polyinosinic-polycytidylic acid (poly(I:C)), a synthetic analog of dsRNA, and a well-known mimic of viral infection.^15^^,^^16^ The NLRP1 inflammasome NLRP1 has recently been reported to sense poly(I:C) as well as Semliki virus dsRNA in keratinocytes.^17^ Here, we found that in NHBE cells, cytosolic delivery of poly(I:C) activated an NLRP1 inflammasome ASC-dependent pathway leading to caspase-1-mediated GSDMD cleavage into a pore-forming fragment. In parallel, poly(I:C) also triggered PKR-dependent caspase-3 activation which both cleaved GSDMD into an inactive fragment incapable of pore formation, and also productively cleaved GSDME into a pore-forming fragment. Overall, both GSDMD and GSDME positively contributed to dsRNA-induced cytokine secretion and cell death. Interestingly, IAV infection activated the PKR but not the NLRP1 pathway to gasdermin pore formation, resulting in PKR-dependent caspase-3-mediated GSDMD inactivation, GSDME pore formation, cytokine secretion, and cell death. Importantly, both GSDMD and GSDME pores were required to limit IAV replication, illustrating an intrinsic antiviral role of gasdermin pores.

## Results

### Cleavage of gasdermins in response to dsRNA sensing in human bronchial epithelial cells

**Figure 1:**
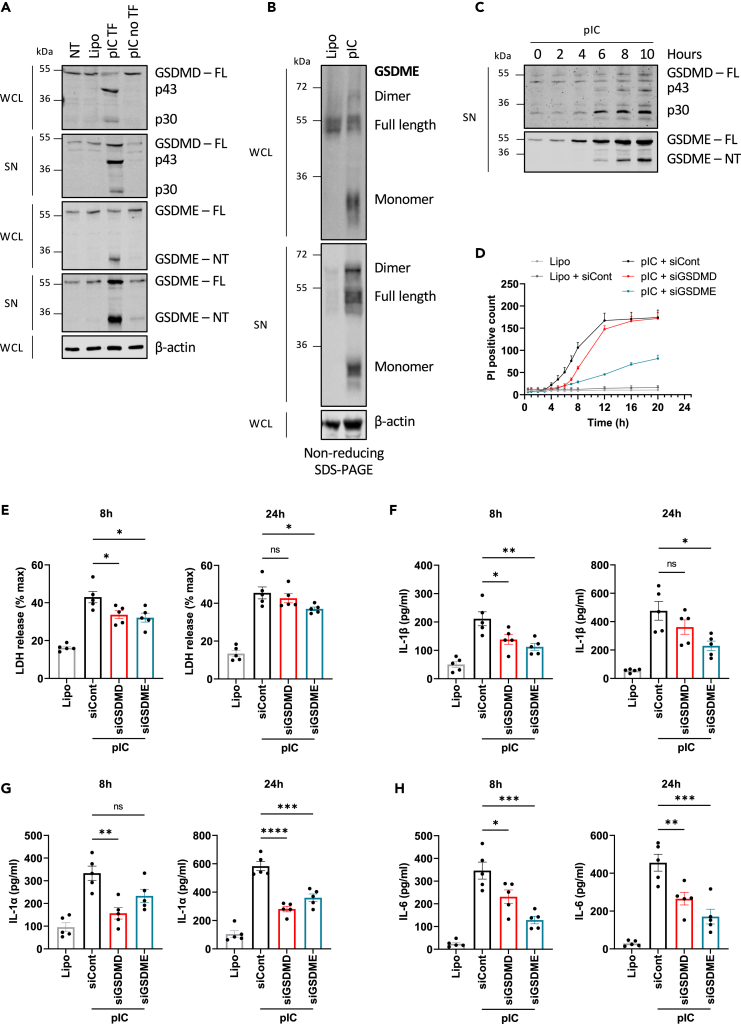
Lytic cell death and cytokine secretion are gasdermin dependent in dsRNA-stimulated NHBE cells (A) dsRNA causes GSDMD inactivation and GSDME activation. NHBE cells were transfected with 2.5 μg/mL poly(I:C) (pIC TF) using lipofectamine, treated with lipofectamine alone (Lipo), or stimulated with non-transfected pIC (pIC no TF) for 16 h. Cell lysates (WCL) and supernatants (SN) were harvested and immunoblotted for GSDMD, GSDME, and β-actin. The appearance of cleaved fragments of GSDMD (p43 and p30) and GSDME (GSDME-NT) is indicated on the immunoblots. (B) GSDME oligomerization in response to dsRNA sensing. NHBE cells were transfected with 2.5 μg/mL pIC for 16 h. Cell lysates and supernatants were harvested, and non-reducing SDS-PAGE was performed to detect GSDME oligomerization. (C) Time course of dsRNA-stimulated GSDMD and GSDME cleavage. NHBE cells were transfected with 2.5 μg/mL pIC. Supernatants were harvested at the indicated times and immunoblotted for GSDMD and GSDME. (D–H) NHBE were transfected with siRNA targeting GSDMD (siGSDMD) or GSDME (siGSDME), or control siRNA (siCont) at 24 and 48 h post-seeding of cells. The following day, NHBE were transfected with 2.5 μg/mL pIC for the indicated times. (D) Time course of PI uptake following pIC stimulation. The number of PI positive cells was analyzed by time-lapse microscopy over a 20 h period. (E) Lytic cell death was assessed by measuring LDH release in the supernatant at 8 and 24 h. (F–H) Secretion of IL-1β (F), IL-1α (G), and IL-6 (H) was quantified by ELISA at 8 and 24 h. Immunoblots are representative of three independent experiments. Data are mean ± SEM of three (D) or five (E–H) independent biological experiments, each performed in triplicate. ns: not significant, ∗p < 0.05, ∗∗p < 0.01, ∗∗∗p < 0.001 and ∗∗∗∗p < 0.0001 by one-way ANOVA. See also Figure S1.

To investigate gasdermin cleavage during dsRNA sensing and RNA virus infection, we initially determined whether dsRNA delivery leads to gasdermin cleavage in primary NHBE cells. Cells were transfected with poly(I:C) for 16 h and whole-cell lysates and cell supernatants were subsequently immunoblotted to detect GSDMD and GSDME fragments. Figure 1A shows that poly(I:C) transfection generated the active p30 pore-forming fragment of GSDMD, but also interestingly a 43 kDa fragment that is known to be indicative of GSDMD inactivation due to alternative non-caspase-1 cleavage. Additionally, poly(I:C) transfection also led to GSDME cleavage, but in this case only the pore-forming GSDME fragment was detected (GSDME-NT, Figure 1A). This pattern of gasdermin processing was not observed with non-transfected poly(I:C), demonstrating that it was due to intracellular sensing of dsRNA. Detection of gasdermin fragments in both the cell lysate and supernatant indicates that poly(I:C) transfection does induce cell lysis. DsRNA-stimulated GSDME activation was unexpected, so in order to confirm that pore-forming fragments were generated, we used non-reducing SDS-PAGE to confirm whether dsRNA-stimulated GSDME fragments could self-assemble into higher protein complexes, which is a surrogate assay for GSDME pore formation.^8^ This was indeed the case, since poly(I:C) transfection led to the appearance of GSDME dimers on the gel (Figure 1B). We also examined the kinetics of gasdermin cleavage following dsRNA transfection and found that GSDMD p30 fragment became prominently visible at 6 h post transfection while the GSDMD p43 fragment was also faintly visible at 6 h post transfection, but was more prominent by 10 h, implying that GSDMD is first activated and then inactivated (Figure 1C). For GSDME, the active pore-forming fragment appeared at 6 h and increased by 10 h (Figure 1C). Together, Figures 1A and 1C imply that in response to cytosolic dsRNA sensing, although initially both GSDMD and GSDME are cleaved into active pore-forming fragments, over time GSDMD is inactivated, while GSDME pore-forming fragments persist.

### DsRNA-stimulated lytic cell death and cytokine secretion are gasdermin dependent

Given that both GSDMD and GSDME are cleaved and that GSDMD is inactivated over time, we next examined the role of gasdermins in propidium iodide (PI) uptake, in order to assess the contribution of GSDMD and GSDME to membrane permeability upon dsRNA sensing. For this, small interfering RNA (siRNA) gene silencing was used to effectively suppress GSDMD and GSDME expression in NHBE cells (Figures S1A and S1B). DsRNA transfection caused PI uptake that plateaued at 12 h, and that was sensitive to gasdermin knockdown (Figure 1D). Up to 8 h, PI uptake was both GSDMD and GSDME dependent, while thereafter PI uptake was mainly dependent on GSDME and not on GSDMD (Figure 1D). This is consistent with GSDMD being inactivated over time, and therefore unable to sustain membrane pores after early time points. Given that gasdermin cleavage leads to pyroptotic cell death,^1^^,^^2^^,^^9^ we next measured lactate dehydrogenase (LDH) release in the supernatant, which is a marker of lytic cell death. Consistent with the PI uptake data, measured up to 20 h, at 8 h, both gasdermins made a small but significant contribution to LDH release (Figure 1E left panel), while at 24 h (4 h later than the final PI uptake time point) only GSDME but not GSDMD contributes to LDH release (Figure 1E, right panel), showing an important role of GSDME in lytic cell death. Besides cell death, GSDMD pores are known to be important for IL-1β secretion downstream of inflammasome activation.^1^^,^^2^ In addition, IL-1β can also be secreted through GSDME pores in macrophages.^8^ To test whether cytosolic dsRNA would elicit IL-1β secretion, and the potential role of gasdermins in this, cells were pretreated with control or gasdermin siRNA prior to transfection of dsRNA, and IL-1β in supernatants measured by ELISA. Figure 1F shows that cytosolic dsRNA did indeed stimulate IL-1β secretion, and that this was inhibited by both GSDMD and GSDME siRNA at 8 h, while at 24 h GSDME but not GSDMD siRNA showed a significant effect on IL-1β secretion. Since GSDMD and GSDME have recently been shown to also be involved in IL-1α secretion in mouse and human macrophages,^7^^,^^18^ and that this cytokine has similar functions to IL-1β, we also considered whether dsRNA would elicit gasdermin-dependent IL-1α release. Indeed, NHBE cells did release IL-1α in response to cytosolic dsRNA sensing, and this cytokine secretion was also inhibited by both GSDMD and GSDME siRNA (Figure 1G). Consistent with these results, IL-6 secretion, which is a downstream consequence of IL-1 release, was also impaired by siRNA-mediated reduction of GSDME expression, but less so by reduction of GSDMD expression (Figure 1H).

Overall, these results show a predominant role of GSDME pores in lytic cell death and cytokine secretion in response to intracellular dsRNA sensing, with a more minor and earlier role for GSDMD.

### Caspase-1 and caspase-3 are required for gasdermin cleavage and cytokine secretion in response to dsRNA

**Figure 2:**
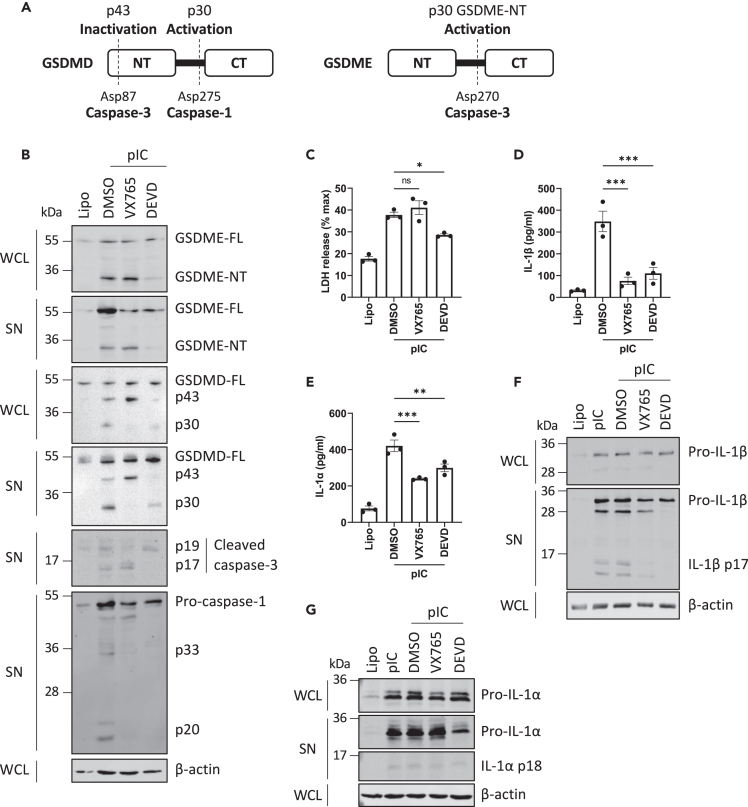
Caspase-1 and caspase-3 are required for gasdermin cleavage and cytokine secretion in response to dsRNA (A) Schematic of caspase-1 and caspase-3 cleavage sites that lead to inactivation or activation of GSDMD and GSDME. (B–G) NHBE cells were treated with DMSO vehicle control, caspase-1 inhibitor (VX765), or caspase-3 inhibitor (DEVD, both 20 μM) for 1 h before transfection of 2.5 μg/mL pIC. (B) Caspase-1 and caspase-3 cleave GSDMD and GSDME. Cell lysates (WCL) and supernatants (SN) were harvested at 6 h and immunoblotted for GSDMD, GSDME, caspase-1, caspase-3, and β-actin. (C) Lytic cell death was assessed by measuring LDH release in the supernatant at 16 h. (D, E) Secretion of IL-1β (D) and IL-1α (E) was quantified by ELISA at 16 h. (F, G) Cell lysates and supernatants were harvested at 16 h and immunoblotted for pro- and mature forms of IL-1β (F) and IL-1α (G), and for β-actin. Immunoblots are representative of three independent experiments. Other data are mean ± SEM of three independent biological experiments, each performed in triplicate. ns: not significant, ∗p < 0.05, ∗∗p < 0.01 and ∗∗∗p < 0.001 by one-way ANOVA.

We next assessed which caspases were activated by dsRNA sensing in order to cleave gasdermins in epithelial cells. The schematic in Figure 2A shows the gasdermin cleavage sites for caspase-1 and caspase-3, and the fragments generated. Since Figure 1 showed that dsRNA sensing caused the appearance of GSDMD and GSDME fragments consistent with caspase-1 and caspase-3 cleavage events, we next assessed the contribution of these caspases to dsRNA-stimulated gasdermin cleavage. Thus, following poly(I:C) transfection, immunoblot analysis was conducted which showed that both caspase-1 and caspase-3 were activated in response to dsRNA sensing, since the caspase-1 and caspase-3 immunoblots show the appearance of the active forms of the caspases (p17 for caspase-3, and p20 and p33 for caspase-1) after poly(I:C) transfection (Figure 2B). We then used the caspase-1 inhibitor VX765 and the caspase-3 inhibitor DEVD to assess the role of these caspases in dsRNA responses. Figure 2B shows that treatment of cells with the caspase-1 inhibitor prior to dsRNA transfection did inhibit caspase-1 activation (6^th^ panel) and appearance of the GSDMD active pore-forming fragment p30 (3^rd^ and 4^th^ panel), but had no effect on GSDME cleavage (1^st^ and 2^nd^ panels). In contrast, the caspase-3 inhibitor did prevent caspase-3 activation (5^th^ panel), and also blocked appearance of the p43 GSDMD fragment (indicative of GSDMD inactivation, 3^rd^ and 4^th^ panel) and appearance of the pore-forming GSDME-NT (1^st^ and 2^nd^ panel). Of note, the caspase-3 inhibitor appeared to also inhibit the appearance of active caspase-1 in the supernatant (Figure 2B, 6^th^ panel), but this was likely due to limiting the secretion of caspase-1, via inhibition of GSDME pore formation.

To test whether caspase-1 and caspase-3 were involved in dsRNA-stimulated lytic cell death and cytokine secretion, LDH was measured in the supernatants of cells treated with the inhibitors. LDH release was only diminished in the presence of caspase-3 inhibitor DEVD and not in the presence of caspase-1 inhibitor VX765 (Figure 2C). These data demonstrate that cell death was caspase-3 dependent and caspase-1 independent. Nevertheless, the addition of either inhibitor reduced IL-1β and IL-1α secretion upon dsRNA treatment (Figures 2D and 2E). This was not due to an effect on pro-IL1β or pro-IL-1α expression (Figures 2F and 2G), indicating that both caspase-1 and caspase-3 are required for secretion of mature IL-1β and IL-1α.

Overall, these results indicate a role for caspase-1 in early GSDMD pore formation and IL-1 secretion, but not cell death, and a role for caspase-3 in GSDMD inactivation, GSDME pore formation, IL-1 secretion, and cell death upon cytosolic dsRNA sensing in epithelial cells.

### DsRNA-stimulated GSDMD cleavage, lytic cell death, and IL-1β secretion are ASC dependent

**Figure 3:**
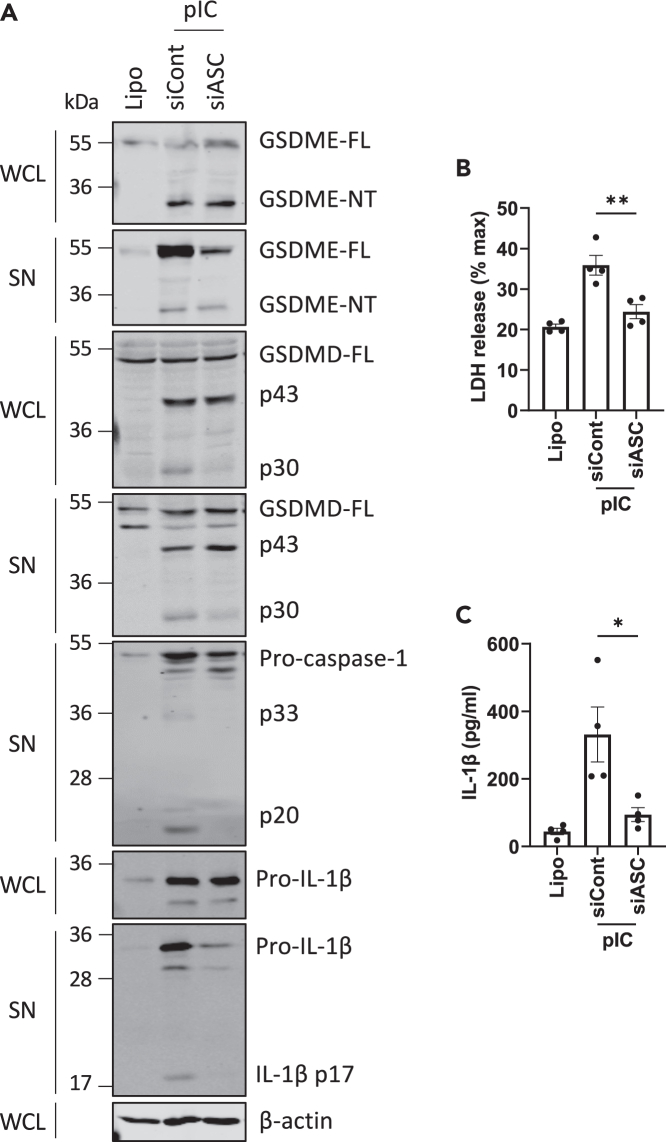
DsRNA-stimulated GSDMD cleavage, lytic cell death, and IL-1β secretion are ASC dependent (A–C) NHBE were transfected with siRNA targeting ASC (siASC) or control siRNA (siCont) using lipofectamine at 24 and 48 h post-seeding of cells. The following day, NHBE were transfected with 2.5 μg/mL pIC for 6 h. (A) GSDMD cleavage is ASC dependent. Cell lysates (WCL) and supernatants (SN) were immunoblotted for GSDMD, GSDME, caspase-1, pro- and mature forms of IL-1β and for β-actin. (B) Lytic cell death was assessed by measuring LDH release in the supernatant. (C) IL-1β secretion was quantified by ELISA. Immunoblots are representative of three independent experiments. Other data are mean ± SEM of four independent biological experiments, each performed in triplicate. ∗p < 0.05 and ∗∗p < 0.01 by unpaired Student’s *t* test. See also Figure S1.

To further investigate the mechanism of caspase-dependent gasdermin cleavage, the involvement of inflammasomes was examined by using siRNA to suppress expression of the inflammasome adaptor ASC (Figure S1C). Figure 3A shows that downregulation of ASC expression prevented dsRNA-stimulated caspase-1 p20 appearance (5^th^ panel) and decreased GSDMD activation (appearance of p30, 3^rd^ and 4^th^ panels), but did not prevent GSDME activation (1^st^ and 2^nd^ panels), nor GSDMD inactivation (appearance of p43, 3^rd^ and 4^th^ panel). In addition, LDH release, as well as IL-1β secretion, was diminished in the absence of ASC (Figures 3B and 3C). Importantly, the reduction in secretion was not due to impaired pro-IL-1β expression (Figure 3A). The requirement for ASC for GSDMD activation, lytic cell death, and IL-1β secretion strongly suggested that dsRNA activated one or more inflammasomes.

### GSDMD cleavage, early lytic cell death, and IL-1β secretion are NLRP1 inflammasome dependent in dsRNA-stimulated epithelial cells

As it has been reported that the RLR adaptor MAVS is involved in dsRNA-stimulated inflammasome activation in some cell types,^19^ the role of MAVS was investigated by suppressing MAVS expression by siRNA (Figure S1D). However, this had no effect on dsRNA-stimulated GSDME or GSDMD cleavage (Figure S2A), nor on lytic cell death (Figure S2B) or IL-1β secretion (Figures S2C and S2D). In addition, we considered whether the NLRP3 inflammasome had a role, since although NLRP3 is mainly expressed in myeloid cells, some studies have shown that NLRP3 inflammasome is activated in respiratory epithelial cells following viral infection,^20^ and also that cytosolic poly(I:C) activated NLRP3 in macrophages.^19^ However, here assessment of the role of the NLRP3 inflammasome revealed the absence of NLRP3 protein expression in unstimulated or poly(I:C)-transfected NHBE cells, in contrast to differentiated human monocytic THP-1 (dTHP-1) cells where it was expressed (Figure S3A). To clarify the role of NLRP3 in responding to intracellular dsRNA in NHBE cells, the NLRP3 inhibitor MCC950 was used. We compared the effect of MCC950 on dsRNA-stimulated NHBE cells to nigericin-stimulated LPS-primed dTHP-1 cells, since nigericin is a known activator of NLRP3. While MCC950 treatment potently blocked both LDH and IL-1β release in nigericin-stimulated LPS-primed dTHP-1 cells, the inhibitor had no effect on these responses to intracellular dsRNA in NHBE cells (Figures S3B and S3C). These data indicate that NLRP3 does not play a role in responding to cytosolic dsRNA in epithelial cells.

**Figure 4:**
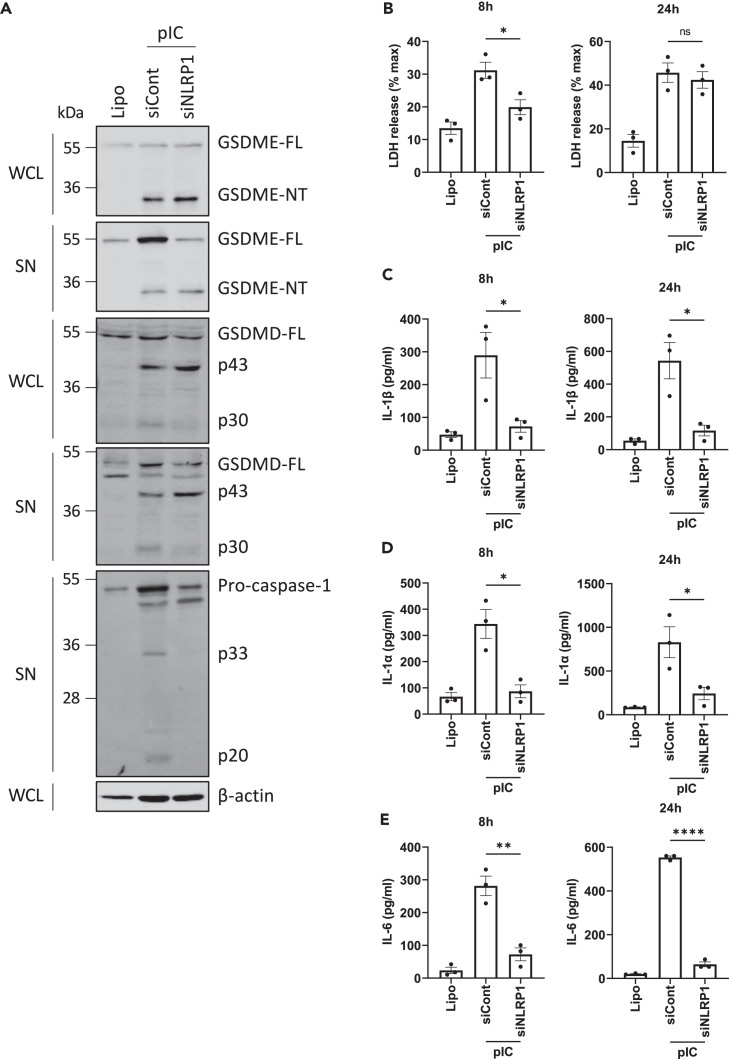
GSDMD cleavage, early lytic cell death, and IL-1β secretion are NLRP1 inflammasome dependent in dsRNA-stimulated epithelial cells (A–E) NHBE were transfected with siRNA targeting NLRP1 (siNLRP1) or control siRNA (siCont) using lipofectamine at 24 and 48 h post-seeding of cells. The following day, NHBE were transfected with 2.5 μg/mL pIC for the indicated times. (A) GSDMD cleavage is NLRP1 dependent. Cell lysates (WCL) and supernatants (SN) were harvested at 6 h and immunoblotted for GSDMD, GSDME, caspase-1, and β-actin. (B) Lytic cell death was assessed by measuring LDH release in the supernatant at 8 and 24 h. (C–E) Secretion of IL-1β (C), IL-1α (D), and IL-6 (E) was quantified by ELISA at 8 and 24 h. Immunoblots are representative of three independent experiments. Other data are mean ± SEM of three independent biological experiments, each performed in triplicate. ns: not significant, ∗p < 0.05, ∗∗p < 0.01 and ∗∗∗∗p < 0.0001 by unpaired Student’s *t* test. See also Figure S1.

Since NLRP1 has recently been described to sense dsRNA in keratinocytes,^17^ we next examined the role of NLRP1 in cleavage of gasdermins in NHBE cells. Interestingly, knockdown of NLRP1 (Figure S1E) did inhibit dsRNA-stimulated caspase-1 activation as the p33 and p20 forms were absent compared to cells treated with control siRNA (Figure 4A, 5^th^ panel), confirming NLRP1 as an intracellular dsRNA sensing inflammasome in NHBE cells. Consistent with this, the caspase-1-dependent activation of GSDMD (appearance of p30, Figure 4A, 3^rd^ and 4^th^ panel) was diminished upon NLRP1 knockdown. However, GSDME cleavage was not impacted by absence of NLRP1 (Figure 4A, 1^st^ and 2^nd^ panel). Further characterization of the role of NLRP1 inflammasome in lytic cell death and cytokine secretion showed that NLRP1 was required for dsRNA-stimulated early lytic cell death (Figure 4B), and for IL-1β, IL-1α, and IL-6 secretion (Figures 4C–4E).

Together, these results define an intracellular dsRNA-stimulated inflammasome signaling pathway in NHBE cells involving NLRP1, ASC, caspase-1, and GSDMD, leading to early lytic cell death and to cytokine secretion.

### DsRNA-stimulated GSDME pore formation, GSDMD inactivation, and caspase-3 activation are PKR dependent

**Figure 5:**
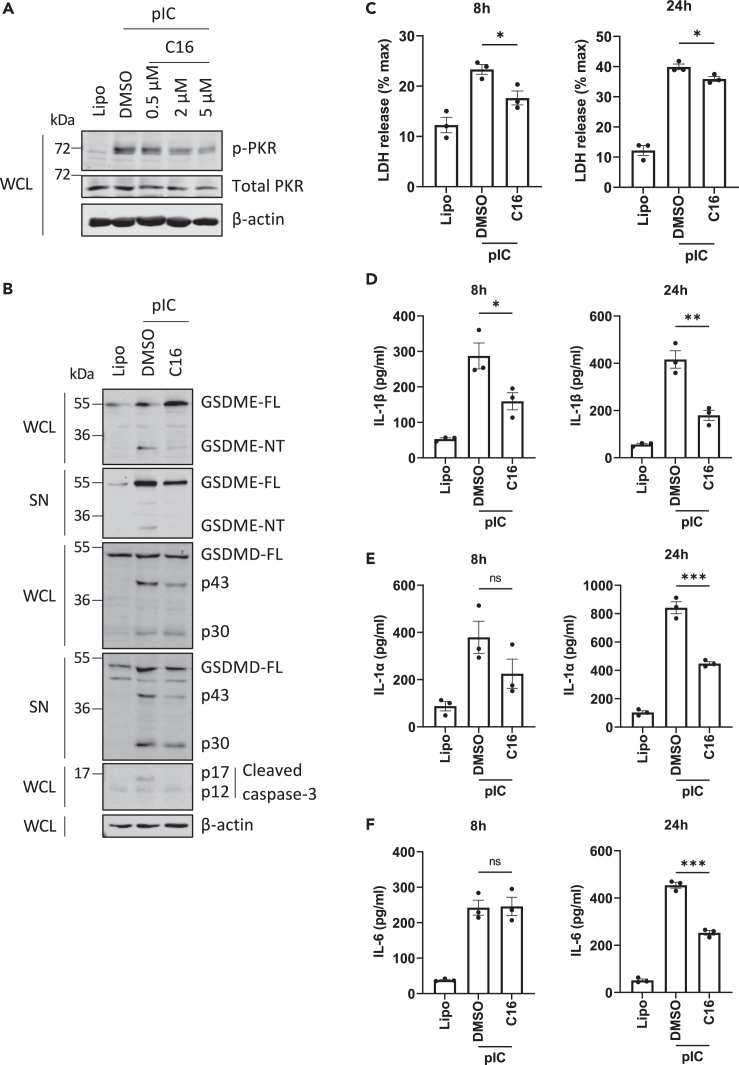
DsRNA-stimulated GSDME pore formation, GSDMD inactivation, and caspase-3 activation are PKR dependent NHBE cells were treated with DMSO vehicle control or PKR inhibitor C16 (2 μM) for 1 h before transfection with 2.5 μg/mL pIC. (A) Inhibition of dsRNA-stimulated phosphorylation of PKR by C16 treatment. Cell lysates (WCL) were harvested at 6 h after treatment and immunoblotted for phospho-PKR (*p*-PKR), total PKR, and β-actin. (B) GSDME activation and GSDMD inactivation are PKR dependent. Cell lysates (WCL) and supernatants (SN) were harvested at 6 h and immunoblotted for GSDMD, GSDME, caspase-3, and β-actin. (C) Lytic cell death was assessed by measuring LDH release in the supernatant at 8 and 24 h. (D–F) Secretion of IL-1β (D), IL-1α (E), and IL-6 (F) was quantified by ELISA at 8 and 24 h. Immunoblots are representative of three independent experiments. Other data are mean ± SEM of three independent biological experiments, each performed in triplicate. ns: not significant, ∗p < 0.05, ∗∗p < 0.01 and ∗∗∗p < 0.001 by unpaired Student’s *t* test.

The data so far demonstrated that upon sensing of intracellular dsRNA, the NLRP1 inflammasome activates GSDMD but not GSDME (Figure 4A) and that MAVS-dependent signaling is not involved in gasdermin cleavage (Figure S2A), in NHBE cells. As translation inhibition triggers caspase-3-dependent GSDME cleavage,^21^ and dsRNA-induced activation of PKR leads to translation inhibition, the role of PKR in GSDME cleavage was investigated. We first confirmed that, in NHBE cells, dsRNA transfection triggered phosphorylation of PKR, a marker of PKR activation (Figure 5A) and that C16, a PKR inhibitor,^22^ suppressed PKR phosphorylation (Figure 5A). We then assessed the ability of C16 to affect dsRNA-stimulated gasdermin cleavage. Figure 5B shows that C16 decreased GSDME cleavage (1^st^ and 2^nd^ panel), and that C16 decreased the appearance of the GSDMD p43 fragment but not the GSDMD p30 fragment (3^rd^ and 4^th^ panel). This indicates that PKR was required for GSDMD inactivation and GSDME activation. Further, PKR inhibition prevented caspase-3 activation (Figure 5B, 5^th^ panel). Thus, dsRNA-dependent PKR stimulation led to caspase-3 activation, which then triggered PKR-dependent GSDME pore formation and GSDMD inactivation. Cognizant with the effects of C16 on GSDME, C16 treatment also inhibited dsRNA-stimulated cell lysis (Figure 5C) and release of IL-1β, IL-1α, and IL-6 (Figures 5D–5F). In conclusion, these data indicate that PKR activation by dsRNA triggers GSDME-dependent lytic cell death and cytokine secretion.

### Caspase-3 and caspase-8, but not caspase-1, mediate gasdermin cleavage after IAV infection

So far, the data indicate that cytosolic dsRNA sensing in NHBE cells leads to GSDMD and GSDME cleavage following NLRP1 inflammasome and PKR activation and that both pores contribute to pyroptosis and cytokine release, although GSDME pores made a greater contribution. We next investigated whether either or both of these pathways toward gasdermin cleavage were involved in sensing of live virus in epithelial cells. For this, we selected IAV, since although being a single-stranded negative-sense RNA virus not expected to produce much viral dsRNA, preliminary experiment showed that IAV infection of NHBE cells gave a similar pattern of gasdermin cleavage as seen for dsRNA. Further, in mouse myeloid cells, IAV has previously been shown to activate GSDME.^23^ However, how inflammasomes and gasdermins might contribute to defense against IAV in epithelial cells is unclear.

**Figure 6:**
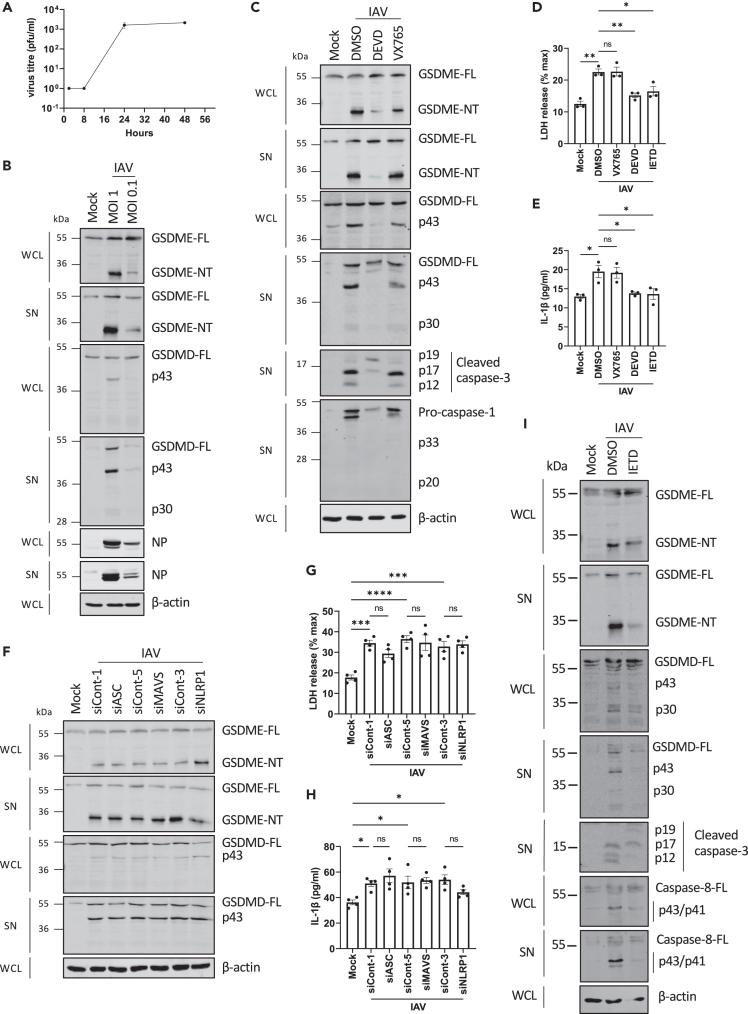
Caspase-3 and caspase-8, but not caspase-1, mediate GSDM cleavage after IAV infection (A) NHBE were infected with IAV WSN at MOI 0.1 and viral titers were determined by plaque assay at 0, 8, 24, and 48 h post infection (hpi). Data are mean ± SEM of two independent biological experiments, each performed in duplicate. (B–I) NHBE were infected with IAV WSN with MOI 1 (and also 0.1 in (B)) for 24 h. (B) Cell lysates (WCL) and supernatants (SN) were immunoblotted for GSDMD, GSDME, IAV nucleocapsid protein (NP), and β-actin. (C–E) NHBE cells were treated with DMSO vehicle control, caspase-1 inhibitor (VX765), caspase-3 inhibitor (DEVD), or caspase-8 inhibitor (IETD, all 20 μM) for 1 h before IAV WSN inoculation. (C) Cell lysates and supernatants were immunoblotted for GSDMD, GSDME, caspase-1, caspase-3, and β-actin. (D) Lytic cell death was assessed by measuring LDH release in the supernatant. (E) IL-1β secretion was quantified by ELISA. (F–H) NHBE cells were transfected with siRNA targeting ASC (siASC), MAVS (siMAVS), or NLRP1 (siNLRP1), or control siRNA (siCont) at 24 and 48 h post-seeding of cells. The following day, cells were inoculated with IAV WSN. (F) Cell lysates and supernatants were immunoblotted for GSDMD, GSDME, and β-actin. (G) Lytic cell death was assessed by measuring LDH release in the supernatant. (H) IL-1β secretion was quantified by ELISA. (I) NHBE cells were treated with DMSO vehicle control or caspase-8 inhibitor (IETD, 20 μM) for 1 h before IAV WSN inoculation. Cell lysates and supernatants were immunoblotted for GSDMD, GSDME, caspase-3, caspase-8, and β-actin. Immunoblots are representative of three independent experiments. Other data are mean ± SEM of three (D, E) or four (G, H) independent biological experiments, each performed in triplicate. ns: not significant, ∗p < 0.05, ∗∗p < 0.01, ∗∗∗p < 0.001 and ∗∗∗∗p < 0.0001 by one-way ANOVA. See also Figure S1.

Upon inoculation of NHBE cells with IAV, viral titers increased over time (Figure 6A) and infection at either an MOI of 0.1 or 1 resulted in intracellular expression of IAV nucleoprotein (Figure 6B), which demonstrates that NHBE cells are permissive for IAV infection. Interestingly, Figure 6B shows that IAV infection caused the activating GSDME cleavage event (1^st^ and 2^nd^ panel), as well as GSDMD inactivation (3^rd^ and 4^th^ panel) with only faint detection of GSDMD p30 at the higher MOI at 24 h post infection (hpi) (4^th^ panel). Upon IAV inoculation, caspase-3 was strongly activated, while the active forms of caspase-1 (p33 and p20) were almost undetectable (Figure 6C). In addition, the caspase-1 and caspase-3 inhibitors were effective at inhibiting their respective caspases after IAV infection (Figure 6C), allowing assessment of the caspase dependency of IAV-stimulated gasdermin cleavage. Immunoblot analysis showed that IAV-stimulated GSDME cleavage and GSDMD inactivation were caspase-3 but not caspase-1 dependent (Figure 6C). However, inhibition of caspase-1 with VX765 did not affect the very moderate GSDMD p30 appearance (Figure 6C, 4^th^ panel), suggesting that caspase-1 was not involved in GSDMD activation during IAV infection. Further, IAV-mediated lytic cell death was caspase-3 dependent but caspase-1 independent (Figure 6D). Given that NHBE cells can secrete both IL-1β and IL-1α following dsRNA delivery (Figures 1F and 1G), we next investigated whether this was the case during IAV infection. Inoculation with IAV elicited a modest elevation in IL-1β secretion (Figure 6E), but not IL-1α (data not shown), and interestingly, IL-1β secretion was dependent on caspase-3 but not on caspase-1 (Figure 6E), even though caspase-1 would be expected to be required for maturation of pro-IL-1β to IL-1β prior to secretion.^24^ Since caspase-1 seemed not to be involved in GSDMD activation or IL-1β secretion, we wondered whether IAV activated a known inflammasome in NHBE cells that would have a role in gasdermin cleavage. Thus, we assessed the effect of silencing ASC, NLRP1, or MAVS expression on IAV responses. Upon IAV inoculation, neither ASC, NLRP1, nor MAVS knockdown affected GSDME activation or GSDMD inactivation (Figure 6F), and IAV-induced lytic cell death was also ASC, NLRP1, and MAVS independent (Figures 6G and 6H). NLRP1 siRNA had a modest, but not significant effect on IL-1β secretion (Figure 6H). These results suggest that IAV-mediated gasdermin cleavage does not require inflammasome activation in NHBE cells.

Next to caspase-1, caspase-8 can also cleave both GSDMD to a pore-forming fragment and pro-IL-1β into its mature form, at the same sites as caspase-1.^25^^,^^26^ Further, IAV can activate caspase-8 in other cell types,^23^ and here we showed that this is also the case in NHBE cells (Figure 6I, 6^th^ and 7^th^ panels). Therefore, we tested whether the caspase-1-independent IAV responses were instead caspase-8 dependent. Inhibition of caspase-8 with the inhibitor IETD (Figure 6I) demonstrated that upon IAV inoculation both LDH and IL-1β release were decreased in the presence of IETD (Figures 6D and 6E), suggesting that caspase-8, but not caspase-1, led to lytic cell death and IL-1β secretion after IAV inoculation of NHBE cells. Further, inhibition of caspase-8 led to a partial suppression of all IAV-dependent GSDMD and GSDME cleavage events, and of IAV-stimulated caspase-3 activation (Figure 6I). Thus, IAV activation of caspase-8 can account for GSDMD activation and contribute to IL-1β secretion and caspase-3 activation.

### IAV infection activates a PKR-caspase-8-caspase-3-GSDME cleavage signaling pathway in NHBE cells

**Figure 7:**
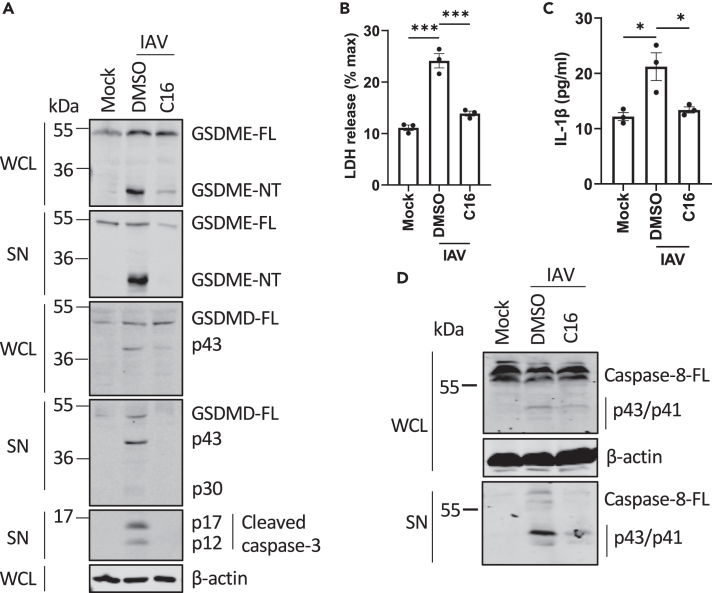
IAV infection activates a PKR-caspase-8-caspase-3-GSDME pathway in NHBE cells (A–D) NHBE cells were treated with DMSO vehicle control or PKR inhibitor C16 (2 μM) for 1 h before IAV WSN inoculation for 24 h. (A, D) Cell lysates (WCL) and supernatants (SN) were immunoblotted for GSDMD, GSDME, caspase-3, caspase-8, and β-actin as indicated. (B) Lytic cell death was assessed by measuring LDH release in the supernatant. (C) IL-1β secretion was quantified by ELISA. Immunoblots are representative of three independent experiments. Other data are mean ± SEM of three independent biological experiments, each performed in triplicate. ns: not significant, ∗p < 0.05 and ∗∗∗p < 0.001 by unpaired Student’s *t* test.

Given that IAV infection, similar to dsRNA, also caused caspase-3-dependent GSDME and GSDMD cleavage (Figure 6C), we wondered whether PKR was also involved in the IAV response. Interestingly, addition of the PKR inhibitor C16 abrogated IAV-mediated GSDME cleavage and GSDMD inactivation and also caspase-3 activation (Figure 7A), indicating that, similar to intracellular dsRNA, IAV utilizes the PKR pathway for gasdermin cleavage. Moreover, LDH and IL-1β secretion in response to IAV inoculation were almost completely prevented when PKR was inhibited (Figures 7B and 7C). Further, IAV-stimulated caspase-8 activation, which we showed was upstream of caspase-3 activation, was also inhibited by C16 (Figure 7D).

Overall, these results demonstrate that IAV infection does not stimulate inflammasome-mediated gasdermin cleavage but rather mainly triggers a PKR-dependent pathway to GSDMD inactivation and GSDME activation involving both caspase-8 and caspase-3.

### Role for gasdermin pores in IAV-induced lytic cell death and cytokine secretion, and in viral replication restriction

**Figure 8:**
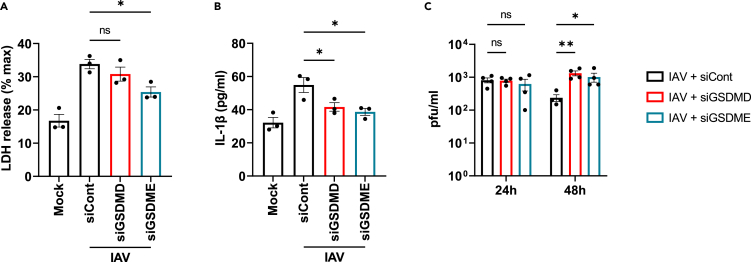
Role for GSDM pores in IAV-induced lytic cell death and cytokine secretion, and in viral replication (A–C) NHBE were transfected with siRNA targeting GSDMD (siGSDMD) or GSDME (siGSDME), or control siRNA (siCont) at 24 and 48 h post-seeding of cells. The following day, cells were infected with IAV WSN at an MOI of 1 (A, B) or 0.1 (C). (A) Lytic cell death was assessed by measuring LDH release in the supernatant at 24 hpi. (B) Secretion of IL-1β was quantified by ELISA at 24 hpi. (C) Viral titers were determined by plaque assay at 24 and 48 hpi. Data are mean ± SEM of three (A, B) or four (C) independent biological experiments, each performed in triplicate (A, B) or duplicate (C). ns: not significant, ∗p < 0.05 and ∗∗p < 0.01 by one-way ANOVA (A, B) or two-way ANOVA (C). See also Figure S1.

Although infections with viruses such as IAV induce gasdermin activation, it is unknown whether gasdermin pore formation is intrinsically antiviral in bronchial epithelial cells. Therefore, to further explore the role of gasdermins in the context of viral infection, the virus-induced lytic cell death and IL-1β release were assessed in cells silenced for GSDMD and GSDME expression using siRNA. While virus-induced lytic cell death was only significantly affected by silencing of GSDME and not GSDMD (Figure 8A), both GSDMD and GSDME pores were required for virus-induced IL-1β secretion (Figure 8B). Interestingly, while suppression of gasdermin expression did not affect virus titers at 24 h post infection, viral titers were higher at 48 hpi in NHBE cells silenced for GSDMD or GSDME compared to cells treated with control siRNA (Figure 8C). Thus, gasdermin pores are important in restricting viral replication in primary human bronchial epithelial cells.

## Discussion

In recent years, much progress has been made in understanding the role of gasdermin proteins in pathogen-induced cell lysis and cytokine release, and in defining the upstream inflammasome pathways that lead to GSDMD pore formation. However, many of the insights have focused on NLRP3-mediated GSDMD activation in myeloid cells, with limited knowledge on how inflammasomes and gasdermins are involved in innate immune responses to pathogens in epithelial cells, even though such cells represent the first line of defense against respiratory RNA viruses such as SARS-CoV-2 and IAV. A major viral PAMP sensed by host cells is dsRNA, and here we defined for the first time two distinct parallel pathways to gasdermin activation that operate in response to dsRNA in primary human bronchial epithelial cells. One pathway, via NLRP1, leads to caspase-1 activation and GSDMD cleavage for pore formation, while the second pathway, via PKR, leads to caspase-3 activation and GSDME cleavage for pore formation, as well as GSDMD inactivation. For dsRNA, the caspase-1-GSDMD pathway contributed to IL-1 secretion and early cell death, while the caspase-3-GSDME pathway was necessary for both IL-1 secretion and sustained cell death.

The finding that dsRNA activates an NLRP1 inflammasome in NHBE cells is consistent with the recent finding that NLRP1 is a dsRNA sensor,^17^ and the emerging literature that NLRP1 is a predominant inflammasome in epithelial cells.^17^^,^^27^ NLRP1 senses viral infection by positive-stranded RNA viruses that replicate in the cytosol, such as Semliki Forest virus and SARS-CoV-2.^17^^,^^27^ Our study indicates that gasdermin and cytokine responses to IAV were not dependent on NLRP1. Thus, IAV responses normally attributed to inflammasome activation in other cells, namely maturation and secretion of IL-1β, and pyroptosis, do not seem to require inflammasome engagement in these cells. Previously, various inflammasomes acting via ASC have been implicated in host defense against IAV, including NLRP3 in mice^11^ and MxA in respiratory epithelial cells,^28^ but in our study, IAV-mediated gasdermin processing was both NLRP1 and ASC independent. Since IAV is a negative-stranded RNA virus that replicates in the nucleus, this may indicate that NLRP1 recognizes cytosolic, positive-stranded RNA viruses, but not nuclear, negative-stranded RNA viruses. Alternatively, IAV may have immune evasion strategies that target NLRP1, but in either case it emphasizes the importance of the PKR-caspase-8-caspase-3-GSDME pathway for sensing of IAV in human epithelial cells to achieve IAV-induced gasdermin pore formation.

Intracellular innate immune sensing of dsRNA involves RLRs in most cell types, while PKR is classically thought of as an effector protein in the antiviral response that functions mainly to phosphorylate eIF2α to inhibit translation, and often only after being upregulated by type I interferon production. However, here we show that RLRs are not involved in intracellular dsRNA- and IAV-stimulated IL-1 secretion and cell lysis, since silencing of the RLR adaptor MAVS had no effect on gasdermin processing, IL-1β release, or cell lysis. Therefore, in epithelial cells, PKR seems to have a more prominent role as a “first responder” to RNA viruses such as IAV than previously appreciated.

That PKR is an important host protein in anti-IAV defense is attested to by the fact that, like many viruses, IAV has evolved to suppress PKR function, in this case via the viral protein NS1.^29^ However, how exactly PKR responds to IAV infection is unclear, since as a nuclear negative-stranded RNA virus, it is not expected to produce substantial amounts of cytosolic viral dsRNA that would directly activate PKR.^30^ What is perhaps more likely is that IAV-induced cell stress activates the PKR-caspase-8-caspase-3-GSDME pathway, for example via direct recognition of excess mitochondrial dsRNA leaking into the cytosol.^31^

The lack of a role for caspase-1 activation in IAV responses raised the question as to how pro-IL-1β was being cleaved into its mature form during IAV infection. Our study in NHBE cells showed that the cleavage of pro-IL-1β upon IAV infection was not due to caspase-1 but to caspase-8 activation, which can cleave both GSDMD and pro-IL-1β, at the same sites as caspase-1.^25^^,^^26^ Further, we showed a role for caspase-8 in IAV-induced lytic cell death. This provided a rationale as to how IAV infection leads to GSDMD activation and IL-1β secretion in the absence of caspase-1 activation, and also suggests that, since caspase-8 activation is downstream of PKR for IAV, the PKR sensing pathway can contribute to all of the IAV responses measured in this study (gasdermin cleavage, cytokine secretion, and cell lysis). Previously, it has been shown that PKR can activate caspase-8.^32^^,^^33^ Another recent study showed that RIG-I sensing of the Zika virus genome activates the extrinsic apoptosis pathway leading to caspase-8 activation, which further mediates caspase-3-dependent GSDME cleavage.^34^ Since translation inhibition leads to caspase-3-dependent GSDME activation in the context of negative-strand VSV infection^21^ and because PKR induces translation shut down, this could suggest that PKR activates caspase-3 through the suppression of protein synthesis in respiratory epithelial cells.

Our results reveal key differences in how primary human epithelial cells, in contrast to other cells previously studied, sense IAV. NLRP3 is known to be involved in antiviral responses to IAV in mouse myeloid cells^35^ while there was no role for NLRP3 here in epithelial cells. Z-DNA-binding protein (ZBP1) is another important antiviral sensor of IAV in both mouse myeloid cells, where it is strongly induced by IAV infection, and in fibroblasts, leading to cell death and inflammatory responses via a RIPK1-RIPK3-caspase-8 axis.^35^^,^^36^^,^^37^ Here, we found that IAV could induce some ZBP1 expression in NHBE cells at a high MOI of 3, but that ZBP1 siRNA had no effect on IAV-stimulated GSDME cleavage (data not shown). Further work will be required to determine what role, if any, ZBP1 has in responses to IAV in human epithelial cells.

It is now well accepted that GSDME pore formation contributes to cytokine release and cell lysis in specific contexts, but different mechanisms of GSDME cleavage have been reported. For example, during SARS-CoV-2 infection, GSDME cleavage is dependent on NLRP1,^27^ whereas we demonstrated that in response to dsRNA and IAV, GSDME cleavage was NLRP1 independent, but PKR dependent. Recently, it has been reported that keratinocytes respond to VSV infection by caspase-3-GSDME-dependent cell lysis, which was dependent on inhibition of protein synthesis,^21^ demonstrating a potentially similar pathway, in a different type of barrier cell, to the inflammasome-independent, PKR-dependent GSDME activation we have identified in NHBE cells.

Whether virus-induced gasdermin pore formation is proviral or antiviral depends on the exact context, with studies to date providing evidence for both possibilities. For instance, IAV H7N9 causes GSDME-dependent pyroptosis leading to a cytokine storm in mouse alveolar epithelial cells.^38^ In addition, during Zika virus infection of mice, GSDME pores caused harmful pyroptosis of placental cells.^34^ Nevertheless, our results in NHBE cells indicate that both GSDMD and GSDME are important to limit IAV replication, and thus in that context are intrinsically antiviral. This is in line with a study showing that Nlrp9b inflammasome-dependent GSDMD-mediated pyroptosis limits rotavirus replication in mouse intestine.^39^ Moreover, an indirect role of GSDME-mediated pyroptosis in restricting viral replication has been illustrated since blocking mitochondrial damage-induced caspase-3-dependent GSDME cleavage in human skin organoids enhanced VSV replication.^21^

In conclusion, our study shows that in NHBE cells both NLRP1 and PKR are sensors of cytosolic dsRNA leading to gasdermin pore formation, cytokine secretion, and lytic cell death. Surprisingly, these cells only require PKR, and not inflammasomes, for IAV-stimulated gasdermin pore formation. This study therefore clarifies how primary human epithelial cells respond to dsRNA and IAV, and highlight an underappreciated role for PKR in antiviral responses in these cells.

### Limitations of the study

We used siRNA to achieve target protein knockdown to reveal the role of signaling proteins and gasdermins in responses to dsRNA and IAV. Although we find that for primary human epithelial cells, siRNA studies give good outcomes, with the advantage of knockdown in real time in real primary cells, CRISPR-Cas9-mediated knockout studies will be needed to further strengthen the conclusions. Also, the primary human bronchial epithelial cells infected with IAV were undifferentiated, so use of differentiated airway-liquid interface cultures would allow further testing of the physiological relevance of the results for human lung.

## STAR★Methods

### Key resources table

**Table:** 

REAGENT or RESOURCE	SOURCE	IDENTIFIER
**Antibodies**		

GSDMD	Sigma-Aldrich	Cat#HPA044487; RRID:AB_2678957
GSDMD	Sigma-Aldrich	Cat#G7422; RRID: AB_1850381
GSDME	Abcam	Cat#ab215191; RRID:AB_2737000
Caspase-1	Adipogen Life Sciences	Cat#AG-20B-0048; RRID:AB_2490257
Caspase-3	Cell signaling	Cat#9661; RRID:AB_2341188
Caspase-8	Cell signaling	Cat#9746; RRID:AB_2275120
IL-1α	PeproTech	Cat#500-P21A; RRID:AB_147560
IL-1β	R&D systems	Cat#AF-201-NA; RRID:AB_354387
ASC	Santa Cruz	Cat#sc-514414; RRID:AB_2737351
MAVS	Santa Cruz	Cat#sc-166583; RRID:AB_2012300
NLRP1	BioLegend	Cat#679802; RRID:AB_2566263
NLRP3	AdipoGen	Cat#AG-20B-0014; RRID:AB_2490202
Phopho-PKR	Abcam	Cat#ab32036; RRID:AB_777310
Total PKR	Santa Cruz	Cat#sc-708; RRID:AB_632247
H1N1 IAV nucleocapsid	Abcam	Cat# ab104870; RRID:AB_10711762
β-actin	Sigma-Aldrich	Cat#A5316; RRID:AB_476743
IRDye 680RD Goat anti-Mouse IgG	Li-Cor	Cat#926-68070; RRID:AB_10956588
IRDye 800CW Goat anti-Rabbit IgG	Li-Cor	Cat#926-32211; RRID:AB_621843
IRDye 800CW Donkey anti-Goat IgG	Li-Cor	Cat#926-32214; RRID:AB_621846

**Bacterial and virus strains**		

Influenza A/WSN/33 (H1N1)	Prof Ron Fouchier (Erasmus MC, The Netherlands)	N/A

**Chemicals, peptides, and recombinant proteins**		

Lipofectamine 2000	Invitrogen	Cat#11668019
High molecular weight poly(I:C)	Invivogen	Cat#tlrl-pic
LPS	Enzo Life Sciences	Cat#581-010-L002
Nigericin	Invivogen	Cat#tlrl-nig
VX-765	Selleckchem	Cat#S2228
Z-DEVD-FMK	MBL	Cat#4800-510
Z-IETD-FMK	MBL	Cat#4805-510
MCC950	Professor Luke O’Neill (Trinity College Dublin, Ireland)	N/A
C16	Sigma-Aldrich	Cat#I9785
Phorbol 12-myristate 13-acetate (PMA)	Sigma-Aldrich	Cat#P1585
Propidium Iodide (PI)	Invitrogen	Cat#P3566

**Critical commercial assays**		

Human IL-1β/IL-1F2 DuoSet ELISA	R&D Systems	Cat#DY201
Human IL-1α/IL-1F1 ELISA	R&D Systems	Cat#DY200
Human IL-6 ELISA	R&D Systems	Cat#DY206
CytoTox 96 Non-Radioactive Cytotoxicity Assay (LDH assay)	Promega	Cat#G1780

**Experimental models: Cell lines**		

NHBE	Lonza	Cat#2540
THP-1	ECACC	ECACC 88081201
MDCK	ECACC	ECACC 85011435

**Oligonucleotides**		

qPCR primers: see Table S1	This paper	N/A
siGSDMD targeting sequence: CAGGAGCTTCCACTTCTACGA	Qiagen	Cat#SI04301556
siGSDME targeting sequence: GCGGTCCTATTTGATGATGAA	Qiagen	Cat#SI00363013
siASC targeting sequence: CGGGAAGGTCCTGACGGATGA	Qiagen	Cat#SI03086783
siMAVS targeting sequence: TTAAAGGAGTTTATCGATGTA	Qiagen	Cat#SI04272702
siNLRP1 targeting sequence: CAGGGTGGAGCTGCATCACAT	Qiagen	Cat#SI00108836
AllStars Negative Control siRNA	Qiagen	Cat#1027281

**Software and algorithms**		

GraphPad Prism 9	GraphPad	Version 9.4.1
Odyssey Software	Li-Cor	Version 3.0.16
Image Studio Lite	Li-Cor	Version 5.2

### Resource availability

#### Lead contact

Further information and requests for resources and reagents should be directed to and will be fulfilled by the lead contact, Andrew G. Bowie (agbowie@tcd.ie).

#### Materials availability

This study did not generate new unique reagents.

### Experimental model and study participant details

#### Primary cells

Primary NHBE cells were purchased from Lonza (CC-2540). The purchased NHBE cells were from two donors, namely a 66-year-old Hispanic Female and a 54-year-old Caucasian Male, and no discernable differences in responses of the two donors to dsRNA nor IAV were observed. All cells were maintained at 37°C in a 5% CO_2_ humidified incubator and were grown until passage 6 in BEBM completed with BEGM bronchial epithelial SingleQuots (Lonza), following the manufacturer’s instructions. Cells were seeded at 30,000 cells per well in 24-well plates and 150,000 cells per well in 6-well plates. The medium was refreshed the day after seeding and cells were stimulated or infected two days later.

#### Cell lines

THP-1 cells (originally from a one-year old male) were purchased from the European Collection of Authenticated Cell Cultures (ECACC) and were cultured in RPMI 1640 + GlutaMAX medium, supplemented with 10% (v/v) FCS, 100 U/ml penicillin, 100 μg/ml streptomycin and 0.1 mg/ml normocin. THP-1 were differentiated with 100 nM of PMA for 48 hours prior to stimulation. Canine MDCK cells were from ECACC and were cultured in DMEM + GlutaMAX medium, supplemented with 10% (v/v) FCS, 100 U/ml penicillin, 100 μg/ml streptomycin and 0.1 mg/ml normocin. All cells were maintained at 37°C in a 5% CO_2_ humidified incubator.

#### Virus strains

Influenza A/WSN/33 (H1N1) was a kind gift from Professor Ron Fouchier (Erasmus MC, The Netherlands). IAV was propagated in MDCK cells and viral titres were determined by hemagglutination assay whereby a 1% suspension of turkey erythrocytes were added to harvested culture supernatants in round bottom plates, and HA patterns were read.^40^

### Method details

#### Cell treatments

Cells were transfected with high molecular weight poly(I:C) (Invivogen, 2.5 μl/ml) using Lipofectamine 2000 (Invitrogen, 1 μl/ml). DMSO vehicle control or VX765, DEVD, IETD (all 20 μM), C16 (2 μM) and MCC950 (25 μM) inhibitors were added one hour prior to stimulation or infection and kept during the whole experiment. For virus infection of NHBE cells, IAV was diluted in BEGM medium and added to the cells for one hour at 37°C with 5% CO_2_. Then, the inoculum was removed, the cells were washed with PBS and fresh BEGM medium was added. Cells were incubated at 37°C in a 5% CO_2_ humidified incubator.

#### IAV replication and titration by plaque assay

Cells were seeded in 6-well plates and infected at MOI 0.1 for one hour. Then, the inoculum was removed, and the cells were washed three times with PBS. 200 μl of supernatant were collected at the time points indicated in the figure legends and stored at -80°C. 200 μl of fresh medium was added to replace the harvested volume. Viral titres were determined by plaque assay. Briefly, 10-fold serial dilutions of harvested supernatants were prepared in duplicate in serum-free DMEM medium. Confluent MDCK monolayers were washed with PBS and 100 μl of the virus dilution was added per well of a 12-well plate for 1 h at 37°C. After 1 h, cells were washed with PBS and overlay medium (0.6 % agarose (Oxoid), 0.7 X MEM, 0.2 % Sodium Bicarbonate, 0.3 % BSA V, 2.8 mM L-Glutamine (all Gibco), 14 mM Hepes, 142 U/ml penicillin, 142 μg/ml streptomycin and 0.007% DEAE-dextran (all Sigma-Aldrich)) was added to the cells. Once agarose was set, plates were placed upside down at 37°C with 5% CO_2_ for 3 days. Then, cells were stained with crystal violet for 20 min and plaques were counted to determine viral titres using the following equation: pfu/ml = mean count of duplicate well x dilution factor x (1000/100).

#### Immunoblotting and GSDME oligomerisation

Cells were scraped in ice-cold PBS and pelleted. Cell pellets were lysed in Laemmli sample buffer, boiled for 5 min at 95°C and subjected to SDS-PAGE and immunoblotting. To visualize GSDME oligomerisation, non-reducing conditions were used by removing DTT from the sample buffer. Proteins in the supernatants were precipitated using strataclean resin (Agilent, 10 μL resin/mL of supernatant) and rotated at 4°C overnight. Supernatants were removed and sample buffer was added. Samples were boiled and subjected to SDS-PAGE and immunoblotting. Nitrocellulose membranes were probed with primary antibodies for either GSDMD (G7422, Sigma-Aldrich 1/1000; HPA044487, Sigma-Aldrich, 1/1000), GSDME (ab215191, Abcam, 1/1000), Caspase-1 (AG-20B-0048, Adipogen, 1/1000), Caspase-3 (9661, Cell signaling, 1/1000), Caspase-8 (9746, Cell Signaling, 1/500), IL-1α (500-P21A, PeproTech, 1/500), IL-1β (AF-201-NA, R&D, 1/1000), ASC (sc-514414, Santa Cruz, 1/500), MAVS (sc-166583, Santa Cruz, 1/500), NLRP1 (679802, BioLegend, 1/500), NLRP3 (AG-20B-0014, Adipogen, 1/1000), phospho-PKR (ab32036, Abcam, 1/1000), total PKR (sc-708, Santa Cruz, 1/500), H1N1 IAV nucleocapsid (ab104870, Abcam, 1/1000) and β-actin (A5316, Sigma-Aldrich, 1/5000). The next day, membranes were incubated with secondary antibodies and analysed using the Odyssey imaging system (LI-COR Biosciences). Images were adjusted using Image Studio Lite ver. 5.2.

#### siRNA gene silencing

Transient siRNA silencing was performed by transfecting siRNA (Qiagen) using Lipofectamine 2000 at both 24 h and 48 h after cell seeding. Medium was refreshed the day after seeding and after the second treatment with siRNA before stimulation or infection. The sequences of the siRNAs used were as follows: GSDMD, 5′-CAGGAGCTTCCACTTCTACGA-3’; GSDME, 5′-GCGGTCCTATTTGATGATGAA-3’; ASC, 5′-CGGGAAGGTCCTGACGGATGA-3’; MAVS, 5′-TTAAAGGAGTTTATCGATGTA-3’; NLRP1, 5′-CAGGGTGGAGCTGCATCACAT-3’. siASC was used at 1 nM, siMAVS at 5 nM and siGSDMD, siGSDME and siNLRP1 were all used at 3 nM. Knockdown was confirmed by measuring mRNA levels by RT-qPCR and protein expression by immunoblot.

#### RNA isolation and RT-qPCR

Total RNA was isolating using High Pure RNA Isolation Kit (Roche) and reverse transcribed with random hexamers (IDT) using Moloney murine leukemia virus reverse transcriptase (Promega) according to the manufacturer’s instructions. The resulting cDNA was analyzed by quantitative RT-PCR using the PowerUp SYBR Green Master Mix (Applied Biosystems) and the following gene-specific primer pairs: *gsdmd*, forward 5′-CAGCACCCTCGCATTCCG-3′, reverse 5′-AGAGAAGGACGTCCAAGTCAGAGTC-3’; *gsdme*, forward 5′- CCAAGACGGTGCAGGTGTCAG-3′, reverse 5′- AGAACTCGAACTGGCCGTCCAG-3’; *pycard* (which encodes for ASC), forward 5′-ATCCAGGCCCCTCCTCAGT-3′, reverse 5′-CGTTTGTGACCCTCGCGATAAGC-3’; *mavs,* forward 5′-GTGCCTACTAGCATGGTGCTC-3′, reverse 5′-GACCCAAGGCCCCTATTCT-3’; *nlrp1*, forward 5′-CTTCAGCAGACGGAAACCAAGTGT-3′, reverse 5′-CCGCCCTCTCTGATCCGA-3’; *gapdh.* forward 5′-TCTTTTGCGTCGCCAGCCGAG-3′, reverse 5′-ACCAGGCGCCCAATACGACCA-3’. Relative mRNA expression was calculated using the comparative C_T_ method, normalizing the gene of interest to the housekeeping gene *gapdh*, and presented as fold induction relative to the control sample set to 1.

#### PI uptake

Propidium iodide (Invitrogen, 1 μg/ml) was added to the cells directly after transfecting poly(I:C). The cell culture plate was then placed in an incubator and the number of PI positive cells was acquired over a 20-hour period using IncuCyte S3 live-cell analysis system (Sartorius).

#### LDH assay

LDH assay was performed using CytoTox 96® Non-Radioactive Cytotoxicity Assay (Promega) according to the manufacturer’s protocol. The absorbance was measured at 490 nm using VersaMax microplate reader (Molecular Devices) and analyzed with SoftMax Pro v7.0.3 software. The data was normalized to untreated lysed cells which are set at 100%.

#### ELISA

Quantification of secreted human IL-1β, IL-1α and IL-6 from cell supernatants was measured by ELISA (R&D, DuoSet ELISA kit) following the manufacturer’s instructions and performed in high binding 96-well plate (Greiner Bio-One).

### Quantification and statistical analysis

All data were analysed with GraphPad Prism 9. Data are presented as mean±SEM. Details of specific statistical tests used (unpaired Student's t test, one-way or two-way ANOVA) are described in figure legends and p values of <0.05 were considered as statistically significant.

## Data Availability

• This paper does not report any original code. • All data reported in this paper will be shared by the lead contact upon request. • Any additional information required to reanalyze the data reported in this paper is available from the lead contact upon request.
