# Supplementary material for: Viral sensing by epithelial cells involves PKR- and caspase-3-dependent generation of gasdermin E pores
Source: iScience. 2023 Aug 21;26(9):107698. doi: 10.1016/j.isci.2023.107698 (PMC10480325; doi:10.1016/j.isci.2023.107698)
Supplement: Document S1. Figures S1–S3 and Table S1 [file mmc1.pdf]

iScience, Volume *26*

Supplemental information

Viral sensing by epithelial cells involves PKR- and caspase-3-dependent generation of gasdermin E pores

Coralie Guy, Marcin Baran, Pau Ribó-Molina, Bernadette G. van den Hoogen, and Andrew G. Bowie

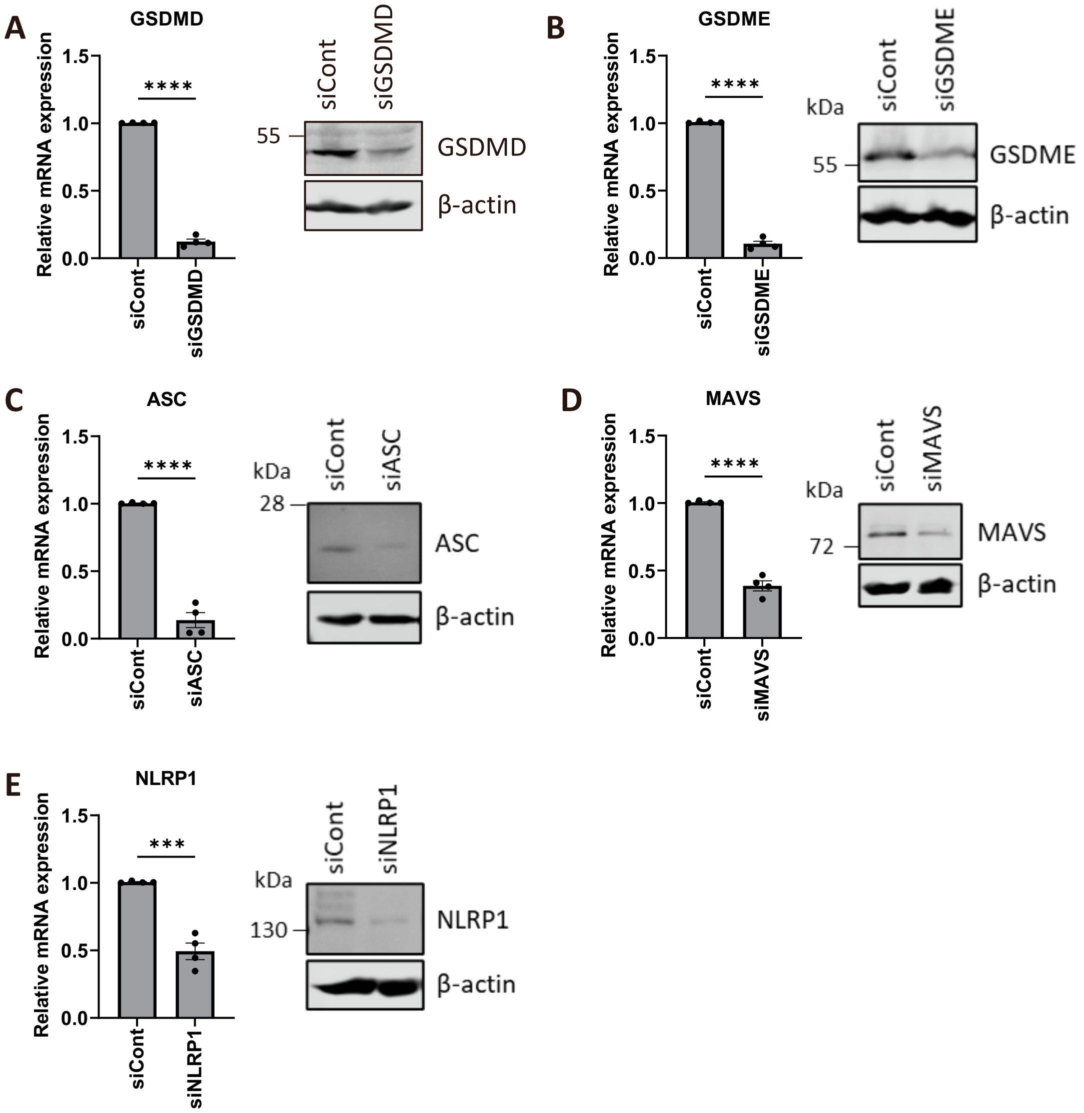

Figure S1. Validation of gene silencing by siRNA in NHBE cells, related to Figures 1, 3, 4, 6 and 8. (A-E) NHBE cells were transfected with siRNA targeting GSDMD (siGSDMD), GSDME (siGSDME), ASC (siASC), MAVS (siMAVS), NLRP1 (siNLRP1) or control siRNA (siCont) at 24 h and 48 h post-seeding of cells. The following day, cells were harvested to confirm the knockdown. GSDMD (A), GSDME (B), ASC (C), MAVS (D) and NLRP1 (E) mRNA expression in the presence of target siRNA relative to control siRNA was quantified by RT-qPCR (left panels), while cell lysates were immunoblotted for the protein of interest (right panels). Immunoblots are representative of three independent experiments. mRNA data are mean±SEM of four independent biological experiments, each performed in triplicate. ***$p < 0.001$ and ****$p < 0.0001$ by unpaired Student's t test.

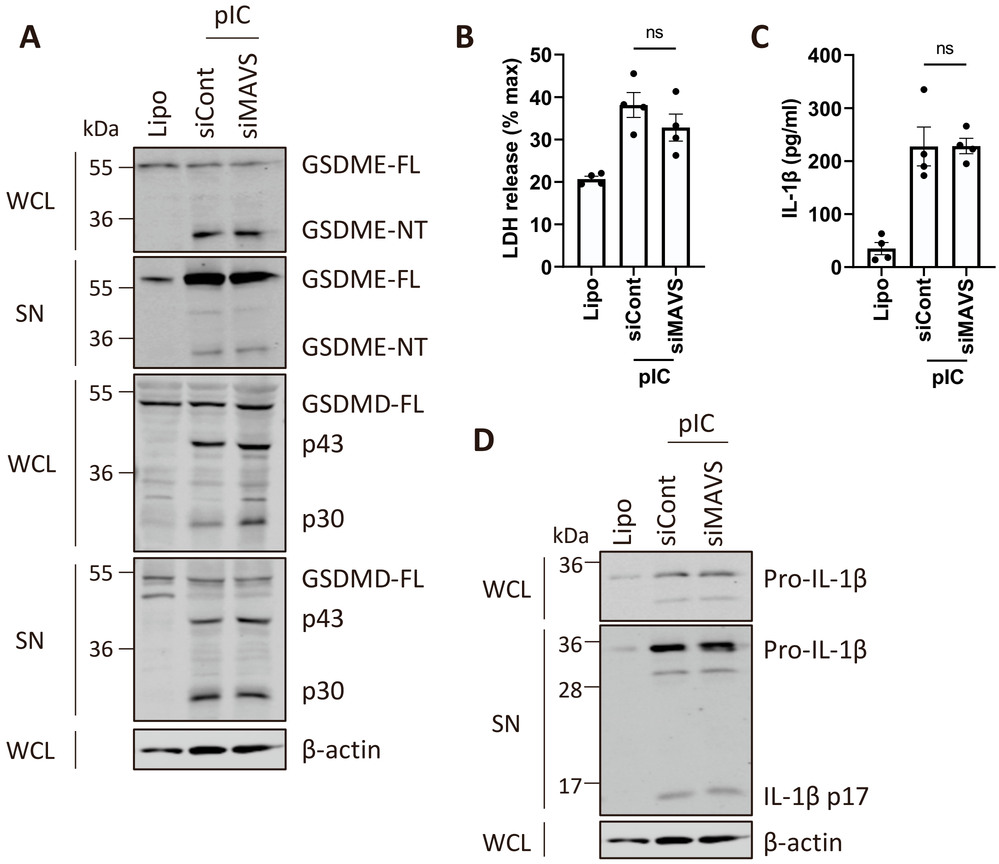

Figure S2. GSDMD and GSDME cleavage is MAVS-independent in dsRNA-stimulated NHBE cells, related to Figure 4. (A-D) NHBE cells were transfected with siRNA targeting MAVS (siMAVS) or control siRNA (siCont) at 24 h and 48 h post-seeding of cells. The following day, NHBE were transfected with 2.5 µg/ml pIC for 6 h. (A) Gasdermin cleavage is MAVS-independent. Cell lysates (WCL) and supernatants (SN) were immunoblotted for GSDMD, GSDME and β-actin. (B) Lytic cell death was assessed by measuring LDH release in the supernatant. (C) IL-1β secretion was quantified by ELISA. (D) Cell lysates and supernatants were immunoblotted for pro- and mature forms of IL-1β and for β-actin. Immunoblots are representative of three independent experiments. Other data are mean±SEM of four independent biological experiments, each performed in triplicate. ns: not significant by unpaired Student's t test.

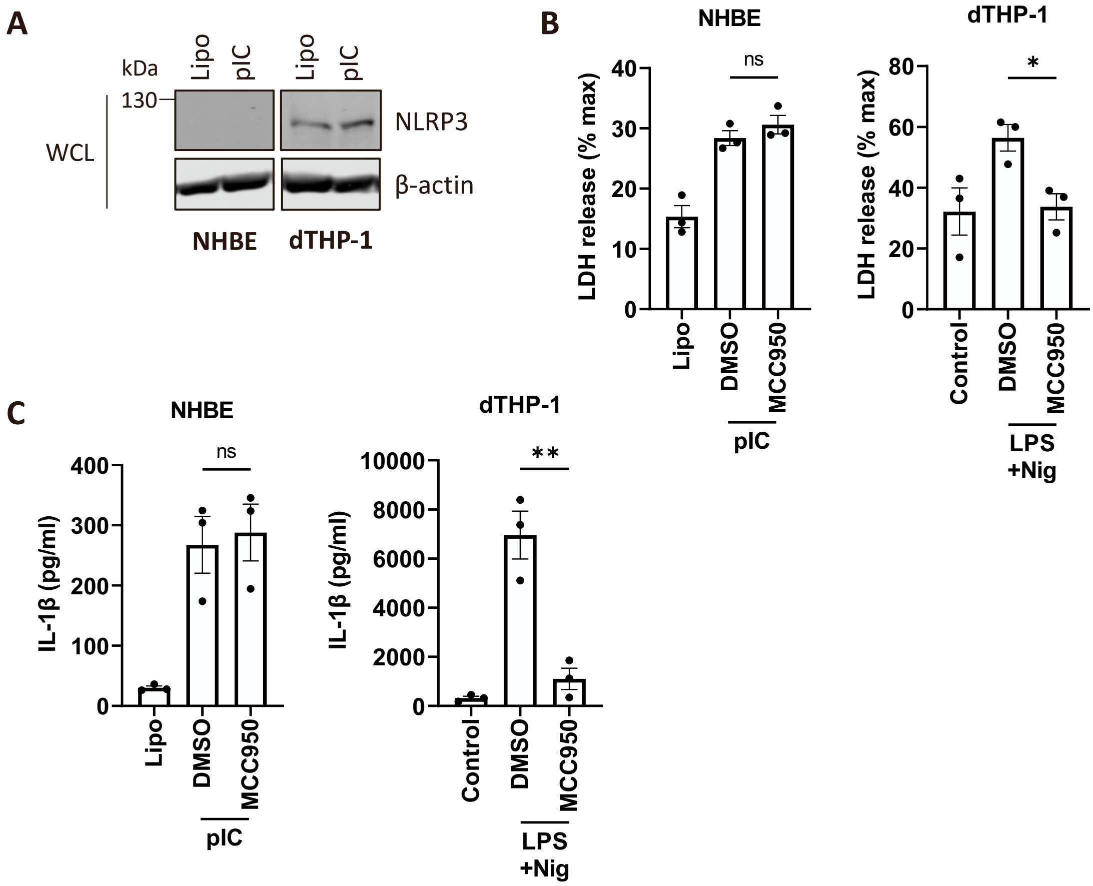

Figure S3. NLRP3 is not required for pyroptosis nor cytokine secretion in dsRNA-stimulated NHBE cells, related to Figure 4. (A) NLRP3 expression is not detectable in NHBE cells. NHBE and dTHP-1 cells were transfected with 2.5 μg/ml pIC for 6 h. Cell lysates (WCL) were immunoblotted for NLRP3 and β-actin. (B, C) NHBE cells were treated with DMSO vehicle control or 25 μM MCC950 for 1 h before transfection of 2.5 μg/ml pIC for 6 h. LPS-primed dTHP-1 were treated with DMSO or 25 μM MCC950 for 1 h before nigericin stimulation for 2 h (10 μM). (B) Lytic cell death was assessed by measuring LDH release in the supernatant. (C) IL-1β secretion was quantified by ELISA. Immunoblots are representative of three independent experiments. Other data are mean±SEM of three independent biological experiments, each performed in triplicate. ns: not significant, *p < 0.05 and **p < 0.01 by unpaired Student's t test.

Table S1. List of primers used for quantitative RT-PCR, related to STAR Methods.

Gene	Sequence 5'-3'
gsdmd (forward)	CAGCACCCTCGCATTCCG
gsdmd (reverse)	AGAGAAGGACGTCCAAGTCAGAGTC
gsdme (forward)	CCAAGACGGTGCAGGTGTCAG
gsdme (reverse)	AGAACTCGAACTGGCCGTCCAG
pycard (ASC) (forward)	ATCCAGGCCCCTCCTCAGT
pycard (ASC) (reverse)	CGTTTGTGACCCTCGCGATAAGC
mavs (forward)	GTGCCTACTAGCATGGTGCTC
mavs (reverse)	GACCCAAGGCCCCTATTCT
nlrp1 (forward)	CTTCAGCAGACGGAAACCAAGTGT
nlrp1 (reverse)	CCGCCCTCTCTGATCCGA
gapdh (forward)	TCTTTTGCGTCGCCAGCCGAG
gapdh (reverse)	ACCAGGCGCCCAATACGACCA
